# Navigating rural medical training: mapping the landscape of resident physician wellbeing using critical realist inquiry

**DOI:** 10.3389/fmed.2026.1792445

**Published:** 2026-03-30

**Authors:** Grace Perez, Aaron Johnston, Rabiya Jalil, Benedicta Antepim, Aliya Kassam

**Affiliations:** 1 Distributed Learning and Rural Initiatives, University of Calgary, Calgary, AB, Canada; 2 Department of Emergency Medicine, University of Calgary, Calgary, AB, Canada; 3 Department of Family Medicine, University of Calgary, Calgary, AB, Canada; 4 People, Culture and Health Promotion, University of Calgary, Calgary, AB, Canada; 5 Department of Community Health Sciences, University of Calgary, Calgary, AB, Canada; 6 Postgraduate Medical Education, University of Calgary, Calgary, AB, Canada

**Keywords:** critical realist inquiry, family medicine, laminated systems, postgraduate medical education (PGME), resident physician, rural health, wellbeing, wellness

## Abstract

**Background:**

Family Medicine residents in rural training programs experience a unique set of challenges and rewards, including professional isolation, resource limitations, and increased autonomy. These contextual features deeply influence their personal and professional wellbeing or *wellness*. The purpose of the study was to map the landscape of wellness resources and initiatives for resident physicians in rural Alberta.

**Method:**

Using a critical realist lens, this study followed a qualitatively-driven sequential explanatory mixed methods design collecting both quantitative (survey) and qualitative (interview) data. Family Medicine residents were invited to complete a survey that collected their demographic data and information about their wellness practices. Follow-up interviews using a semi-structured guide were conducted with residents and preceptors in rural clinical sites. Interviews were coded by at least two members of the research team independently. Discrepancies in coding were discussed until consensus was achieved. The context, mechanism, and outcome (CMO) configurations were applied to Bhaskar’s laminated systems model (LSM) to describe the interpretation and integration of both data sources.

**Results:**

27 rural family residents participated in an online survey, with 7 participating in semi-structured interviews. An additional 7 rural preceptors were interviewed to examine resident wellness from their perspective. Most residents reported positive experiences and adequate support from their programs. However, frequent stress and burnout remained a concern for over one-third of residents. Community integration and participation was reported as integral to maintaining wellness for residents, with preceptors leveraging mentoring relationships as a way to support resident wellness.

**Conclusion:**

The findings allow medical educators to recognize the systemic and relational conditions that promote resident wellness and support resilience. This could potentially encourage the design of interventions that are attuned to the layered structure of reality, targeting not only individual coping strategies (e.g., self-care) but more importantly structural and systemic reforms, institutional continuity, and relational support systems.

## 1 Introduction

The training of physicians intending to practice in rural areas is highly relevant to addressing healthcare disparities in underserved areas but faces unique challenges (1). The transition from a learner mindset to a professional medical identity can be perplexing and require significant adjustment (2). In Canada and internationally, resident physicians (hereon referred to as residents) face day-to-day challenges like demanding workload, long work hours, high levels of stress and responsibility, navigating complex healthcare systems, and balancing education and interpersonal dynamics (3, 4). In addition, financial strain from low salaries and student loans, and uncertainty about future career path (5) can contribute to burnout and mental health crisis, prompting concern for resident wellbeing, also referred to as *wellness*. Issues relating to wellbeing are widely discussed in medical education and the problem of poor wellbeing in residency has been established (6). Burnout, depression, and suicidal ideation are prevalent among resident physicians at higher rates than their non-physician peers and with these issues persisting into practice (7–9). This takes a toll on residents, who are in formative stages of their lives and careers, with consequential impact on patient care. Compromised trainee wellbeing has been linked to diminished function, reduced empathy, poorer quality of care, and higher medical error rates (10–13). Despite increased awareness and recognition of the prevalence of physician burnout and the associated risks of depression and suicide, there is a lack of actionable guidelines for residency programs to mitigate these risks for their residents (14, 15).

Family Medicine residents in rural training tracks experience a unique set of challenges and rewards, including professional isolation, resource limitations, and increased autonomy (16). These contextual features deeply influence not only their clinical education but also their personal and professional wellbeing. Public health emergencies, such as the SARS outbreak and the COVID-19 pandemic, introduced a new set of pressures and disruptions that further complicated rural medical education, affecting preceptors and trainees alike (17, 18). The COVID-19 crisis severely impacted practice-based learning, causing disruptions to learning environments in healthcare facilities and limiting the acquisition of new clinical skills (19–21), while the earlier SARS outbreak similarly disrupted residency training, leading to altered training environment, a loss of formal educational sessions, and increased stress from rigorous isolation procedures in emergency departments (22). Moreover, it is unclear how resident wellbeing manifests in community training settings which may impact both learner and patient safety. On the one hand, distance can operate as a positive influence for residents and programs fostering resilience. Resilience, however, depends on social capital, such as communities of practice among the local population of patients, health care providers, residency, and faculty. On the other hand, the small size and remoteness of rural residency may make trainees vulnerable to stress, and a lack of policies and procedures (23). In the province of Alberta, Canada, there is a paucity in rural resident wellness initiatives, stemming from a lack of understanding of how rural residency programs facilitate wellness for their residents and how rural residents themselves achieve wellness can lead to difficulties in the development, implementation, and sustainability of initiatives.

Understanding the lived realities of rural Family Medicine residents, particularly during a global crisis, is critical for designing supportive training environments (24). The authors of this study are from a medical school in Western Canada and aimed to map the landscape of wellness resources and initiatives for resident physicians in rural Alberta. Using a critical realist lens (25, 26), the authors sought to explore how rural track residents experience wellbeing during training, including the impact of the COVID-19 pandemic, to understand what wellness resources and services are available for rural resident physicians, and how residents accessed and made use of these resources. Ultimately, the goal is to identify modifiable barriers to wellness for residents to inform recommendations for the implementation of rural resident wellness initiatives, which may help foster the collective capacity of rural medical trainees in Alberta.

## 2 Methods

### 2.1 Theoretical framework

#### 2.1.1 Philosophical orientation

This study draws on a critical realist approach (25, 26), guided by Bhaskar’s critical realist lens (27–29), to explore the generative mechanisms shaping rural track Family Medicine resident wellbeing during training and educational experiences in rural contexts. Critical realism allows research to go beyond describing experiences to theorizing the causal structures that generate them. The study inquiry focuses on the relationships between context (C), mechanisms (M), and outcomes (O). This is applicable to both qualitative and quantitative research methods, and data may be drawn from the literature or from empirical sources (or both) to test the philosophical theory to explain why a phenomenon works (25).

More specifically, Bhaskar posits a laminated systems model (LSM) to understand the layered nature of reality, as existing in layers or levels that interact with each other (30, 31). This approach suggests that social phenomena are not isolated events but rather emerge from and are influenced by multiple levels of interaction (32):

(i) Sub-individual: physiological aspects of individuals
(ii) Individual: psychological aspects of individuals
(iii) Micro: small groups or populations
(iv) Meso: functional roles and relationships within organizations or communities
(v) Macro: large-scale societal structures and dynamics
(vi) Mega: civilizations and traditions
(vii) Global/Planetary: global issues and their impact on the planet

**Figure 1:**
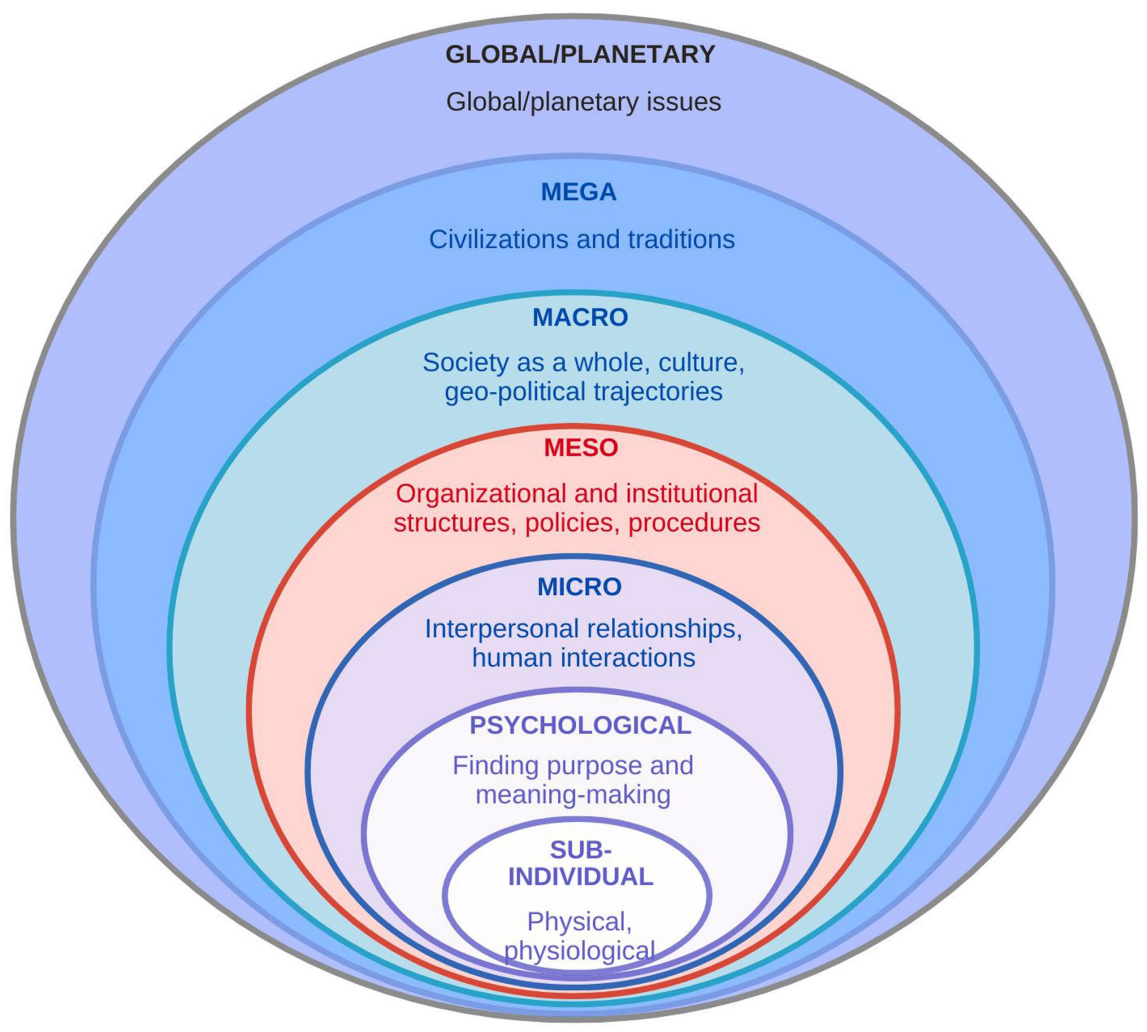
Diagram of Bhaskar’s stratified domains of reality laminated systems model.

The model emphasizes the interconnectedness layered nature of reality, with different levels of analysis influencing each other (Figure 1), with the individual being at the center and spreading outwards to external global and planetary issues. The critical realist lens is particularly valuable in the context of medical training, where wellbeing is shaped by individual, institutional, community, and both systemic and structural factors, many of which are latent or obscured. This lens allows for the examination of complex and multi-layered experiences of physician trainees, especially during an unprecedented disruption such as the COVID-19 pandemic. This approach supports an inquiry into not only what happened to rural residents during training but why and how those experiences came to be shaped by deeper structures. In essence, Bhaskar’s critical realism provides a framework for understanding the complex and layered nature of reality, particularly in social contexts, by emphasizing the interplay between different levels of analysis and the importance of causal mechanisms (33).

#### 2.1.2 Evaluation approach

Seeking to understand the state of the physician trainee wellbeing in rural family medicine residency training settings, the study team employed a ‘thinking with the theory approach’ (34–37) and applied Bhaskar’s critical realist lens to all aspects of the study (27–29). This multi-layered approach is appropriate in this research as it emphasizes that there are many underlying mechanisms that interact and influence each other, creating an interconnected structure.

**Figure 2:**
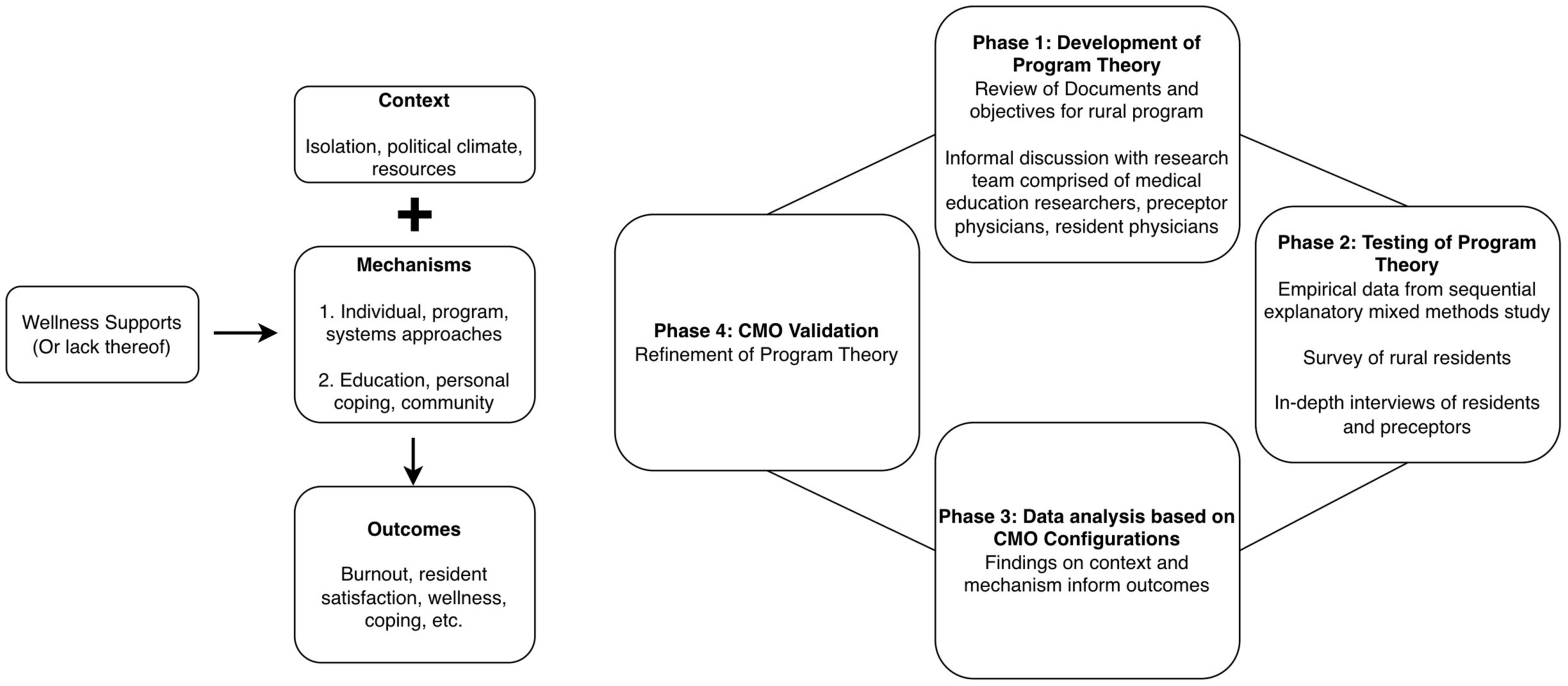
Initial program theory (left) and phases of the study (right).

**Figure 3:**
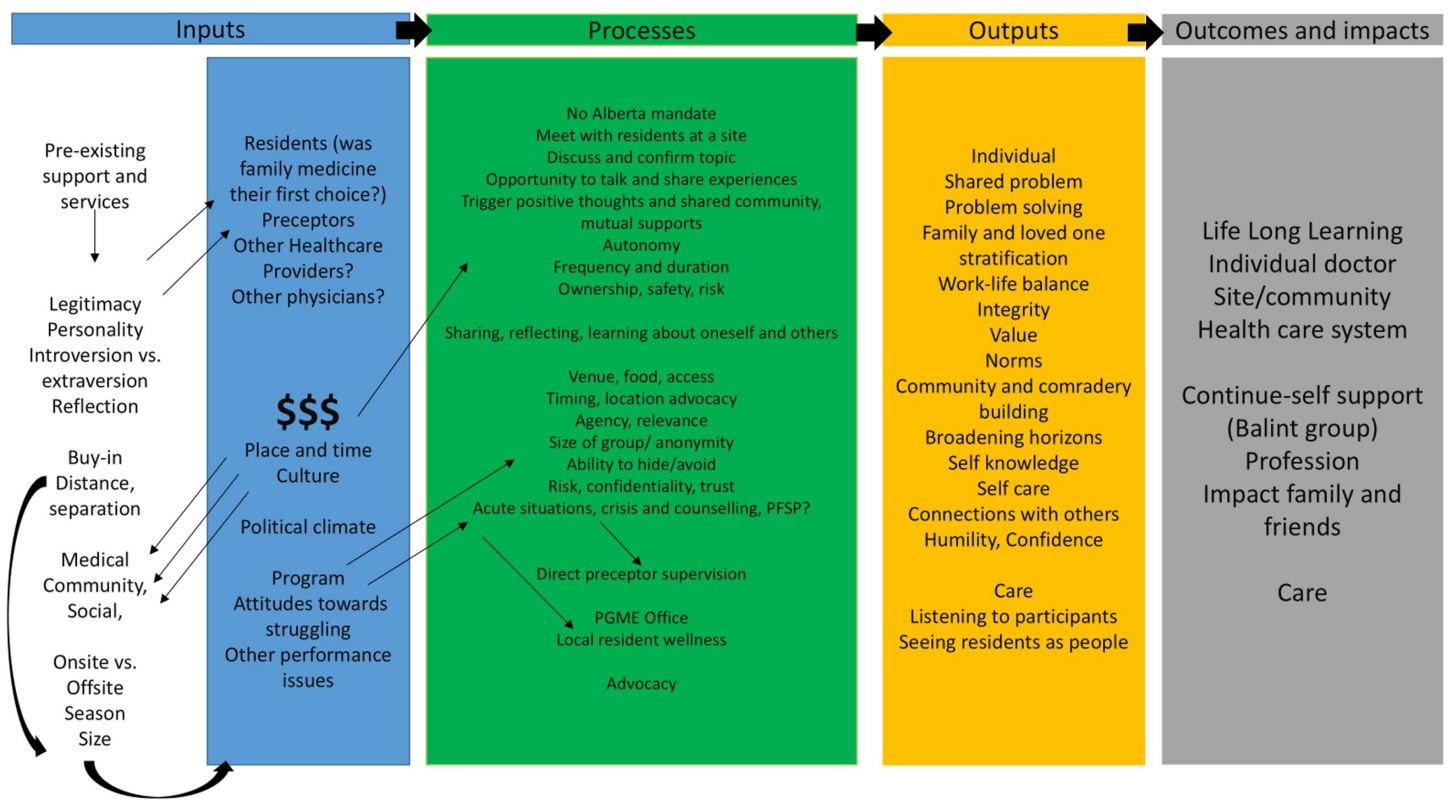
Phase 1—development of the programmatic theory.

This evaluation was conducted in three research phases: (1) development of programmatic theory (PT) underlying rural resident wellness; (2) testing of PT using empirical data; and (3) refinement of PT. Figure 2 shows the initial program theory and phases of the research, while Figure 3 shows the development of the programmatic theory. Programmatic theory was developed through reviews of rural residency rotation objectives, documents, and informal discussions with key stakeholders such as program directors, and faculty members in rural family medicine (Figure 3). These stakeholders were identified and recruited through publicly available contact information on institutional websites. Then, a sequential explanatory mixed methods study was conducted to assess context (C), mechanisms (M), and outcomes (O) to test the PT. The survey data was collected to inform the interview data and together comprise our evaluative interpretation.

### 2.2 Study design

In phase 2 of the study, the C-M-O evaluation phase, followed a sequential design to explore experiences of Family Medicine residents in rural training track to uncover the contextual, structural, and agential mechanisms of shaping resident wellbeing during the pandemic. The design was informed by the realist evaluation framework (26), aligned with Bhaskar’s laminated systems. This approach is especially suitable for exploring how complex contextual factors, such as rurality, healthcare system structures, and disruptive events such as the COVID-19 pandemic, interact with individual experiences of wellbeing among rural family medicine residents.

#### 2.2.1 Setting and participants

The study was conducted across rural Family Medicine rotation sites at the University of Calgary and University of Alberta, among rural residents and preceptors in the rural program of the Department of Family Medicine.

### 2.3 Data collection

Data collection took place between 2020 and 2022 and was analyzed from 2023 to 2025. Quantitative and qualitative data were collected. Family Medicine residents were invited to complete a survey that collected their demographic data and information about their wellness practices, including concerns and coping strategies, and intentions to live in a rural setting. Follow-up interviews using a semi-structured guide were conducted with consenting physician trainees to investigate their wellness and wellbeing while on rural placement. Interviews were performed and recorded over the Zoom platform. In addition, faculty members and staff who were identified as preceptors in the rural rotation sites were also invited to take part in the study and were interviewed upon consent.

Ethical approval was obtained from the University of Calgary Conjoint Health Research Ethics Board (REB20-0438). Participants provided informed consent and were reminded of their right to withdraw at any time. Data confidentiality and participant anonymity were strictly maintained.

### 2.4 Data analysis

#### 2.4.1 Quantitative data

Quantitative data from the online survey were cleaned before further analysis using SPSS (version 29.0). Participants’ characteristic data were summarized using descriptive statistics. The attitude and perception scores about rural medical practice and experiences of residents during training were reported in terms of counts and percentages and were presented graphically as well.

#### 2.4.2 Qualitative data

The authors performed reflexive thematic analysis (38, 39) on qualitative data from resident and preceptor participants, to understand the lived realities of rural family medicine residents and preceptors through the laminated systems model. The analysis of these different levels is crucial to gain a better understanding of the wellness of rural Family Medicine residents. It enabled the study team to explore the multifaceted experiences of wellness and wellbeing among medical trainees in rural settings.

While Bhaskar often discusses a seven-level system, the authors propose a condensed model of social being, which includes (i) the individual/psychological (personal), (ii) micro, (iii) meso, (iv) macro, and (v) global/planetary levels. The *personal level* focuses on the individual’s personal actions, experiences, and perceptions. The micro (*interpersonal or relational*) level examines small groups, interactions, and social practices. The meso level (*organizational or institutional*) explores the relationships between functional roles, social structures, and institutions. The macro (*cultural/societal*) level analyzes the functioning of larger social systems, such as societies or civilizations. The *global or planetary level* relates to global events and planetary issues. This framework helps analyze resident wellbeing by considering the interplay between these different levels of social organization.

### 2.5 Reflexivity and positionality

The research team consisted of two physicians experienced in educational leadership (AJ, RJ), a wellness and wellbeing expert (AK) and two research staff who are adept in qualitative research (GP, BA). AJ is the associate dean for distributed learning and rural initiatives, overseeing all distributed teaching activities. RJ is the associate dean of people, culture and health promotion, which includes supporting student wellness and initiatives on campus. AK is internationally recognized researcher in measuring stigma and discrimination toward mental illness and is the founder of the learner wellness laboratory, Wellness Innovation Scholarship for Health Professions Education and Sciences (WISHES) (40). GP supports the various scholarship activities of the distributed teaching faculty, preceptors, and medical learners.

## 3 Results

### 3.1 Study participants

A total of *N* = 27 rural physician trainees completed the online survey. The demographic profiles of the resident survey participants are summarized in Table 1. The ages of the residents ranged from 23 to 43 years old, with an average of 30 years. Most (81%) were white, 63% identified as a woman and about 60% were single or unpartnered. Majority (70%) have grown up in a rural community. Of the 27 residents who completed the survey, 7 also participated in semi-structured interviews – 4 from University of Calgary and 3 from University of Alberta, and 7 preceptors, and 4 women and 3 men. In addition, 7 preceptors were also interviewed, 4 women and 3 men, each one representing a different rural community.

**Table 1:** Demographic profile of rural family medicine residents.

Resident demographics		N	%
Participated in the study		27	100
Institution	University of Calgary	12	44
	University of Alberta	12	44
	Not specified	3	11
Year in FM residency program	Year 1	15	56
	Year 2	12	44
Age	Mean (SD)	29.79 (3.75)	
	Range	(23, 43)	
Gender	Identifies as a man	10	37
	Identifies as a woman	17	63
Racial identity	White	22	81
	Black, Indigenous, Person of Color	5	19
Marital status	Single	15	56
	Partnered	10	37
	Divorced	1	4
Children	Yes	5	19
	No	22	81
	Prefer not to disclose	1	4
Grew up in a rural community	Yes	19	70
	No	8	30

### 3.2 Experiences during residency and attitudes toward rural medical practice

**Figure 4:**
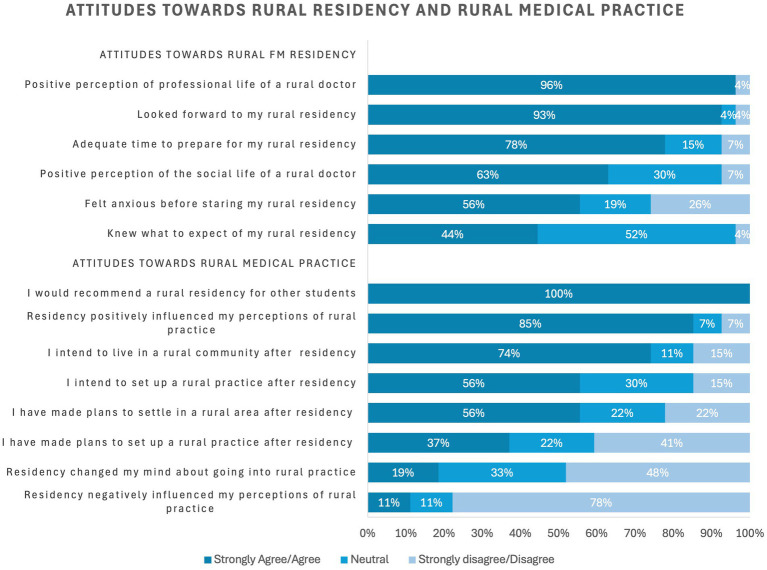
Attitudes toward rural FM residency and rural medical practice.

**Figure 5:**
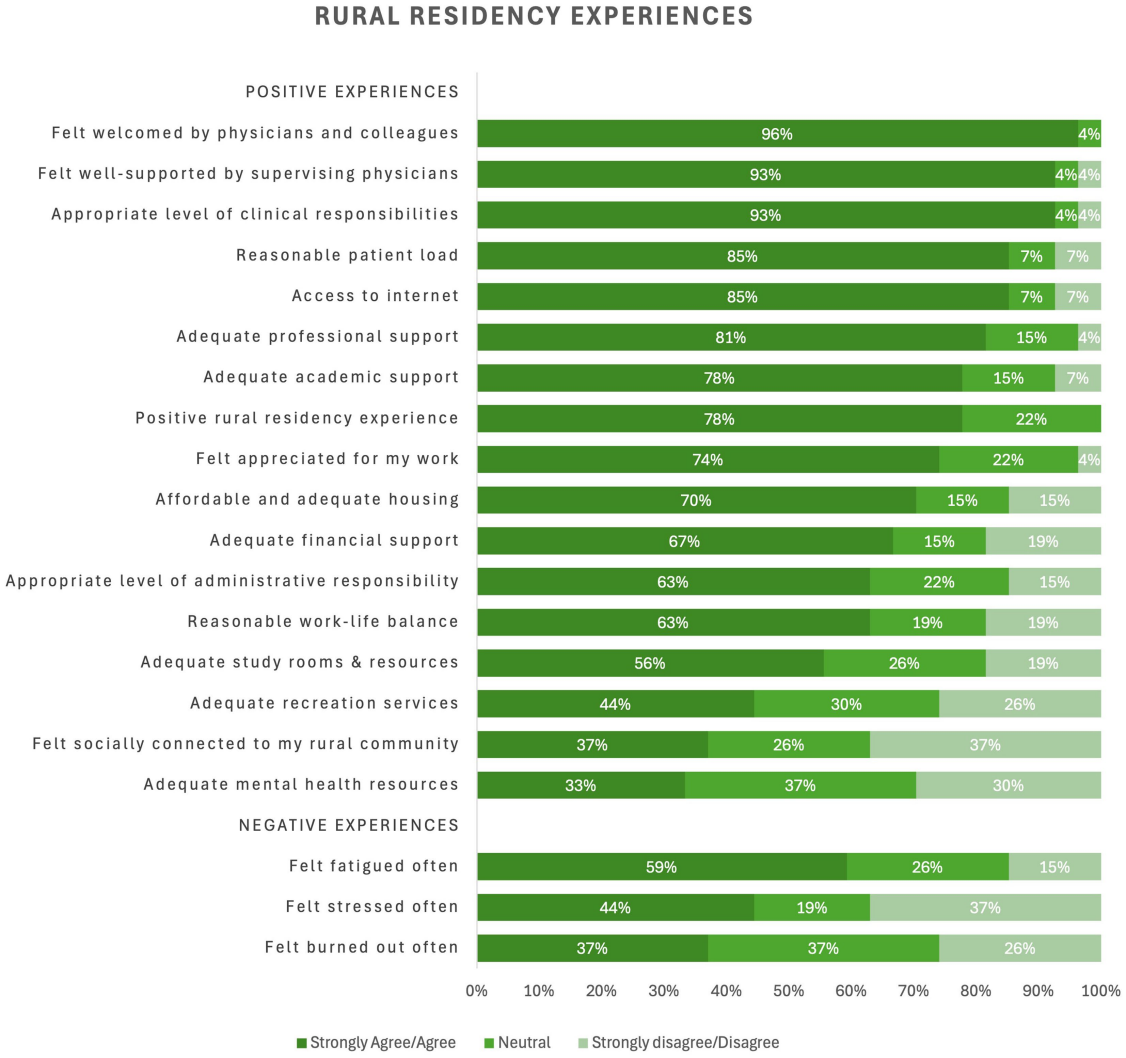
Experiences during rural FM residency.

Presented in Table 2 and Figures 4, 5 are the participant ratings of the various attributes of rural Family Medicine residency training. Overall, perceptions about the Family Medicine residency experience were positive. All participants (100%, *n* = 27) indicated that they would recommend a rural residency to other students. They also reported a highly positive perception of the professional life of a rural physician (96%, *n* = 26) and most looked forward to their rural training (93%, *n* = 25). After residency, 85% (*n* = 23) said that their residency positively influenced their perception of rural practice, so much so that 74% (*n* = 20) intended to live in a rural community to practice medicine.

**Table 2:** Experiences about rural family medicine residency and attitudes toward rural medical practice.

Rural FM residency attitudes and experiences	Strongly disagree	Disagree	Neutral	Agree	Strongly agree
Attitudes toward rural residency					
I felt anxious before staring my rural residency	4 (15%)	3 (11%)	5 (19%)	12 (44%)	3 (11%)
I was looking forward to my rural residency	0 (0%)	1 (4%)	1 (4%)	16 (59%)	9 (33%)
I knew what to expect of my rural residency	0 (0%)	1 (4%)	14 (52%)	10 (37%)	2 (7%)
I had adequate time to prepare for my rural residency	1 (4%)	1 (4%)	4 (15%)	17 (63%)	4 (15%)
I had a positive perception of professional life as a rural physician	(0%)	1 (4%)	0 (0%)	17 (63%)	9 (33%)
I had a positive perception of the social life of a rural physician	1 (4%)	1 (4%)	8 (30%)	13 (48%)	4 (15%)
Rural residency experiences					
I received adequate financial support	0 (0%)	5 (19%)	4 (15%)	11 (41%)	7 (26%)
I had access to affordable and adequate housing	(0%)	4 (15%)	4 (15%)	13 (48%)	6 (22%)
I had an appropriate level of clinical responsibilities	(0%)	1 (4%)	1 (4%)	18 (67%)	7 (26%)
I received adequate academic support	(0%)	2 (7%)	4 (15%)	12 (44%)	9 (33%)
I felt burned out often	1 (4%)	6 (22%)	10 (37%)	5 (19%)	5 (19%)
I had access to adequate study resources and study rooms	(0%)	5 (19%)	7 (26%)	11 (41%)	4 (15%)
I had adequate access to internet	(0%)	2 (7%)	2 (7%)	15 (56%)	8 (30%)
I received adequate professional support	(0%)	1 (4%)	4 (15%)	15 (56%)	7 (26%)
I had a reasonable patient load	(0%)	2 (7%)	2 (7%)	18 (67%)	5 (19%)
I felt welcomed by physicians, nurses and other staff at my rural site	(0%)	(0%)	1 (4%)	12 (44%)	14 (52%)
I felt well-supported by supervising physicians	1 (4%)	0 (0%)	1 (4%)	16 (59%)	9 (33%)
I felt stressed often	1 (4%)	9 (33%)	5 (19%)	8 (30%)	4 (15%)
I felt socially connected to my rural community	(0%)	10 (37%)	7 (26%)	9 (33%)	1 (4%)
I had access to adequate recreation services in my rural community	(0%)	7 (26%)	8 (30%)	10 (37%)	2 (7%)
I had access to adequate mental health resources	(0%)	8 (30%)	10 (37%)	8 (30%)	1 (4%)
I had a reasonable work-life balance	1 (4%)	4 (15%)	5 (19%)	16 (59%)	1 (4%)
I felt fatigued often	(0%)	4 (15%)	7 (26%)	9 (33%)	7 (26%)
I had an appropriate level administrative responsibility	1 (4%)	3 (11%)	6 (22%)	15 (56%)	2 (7%)
I felt appreciated for my work	(0%)	1 (4%)	6 (22%)	15 (56%)	5 (19%)
I had a positive rural residency experience	(0%)	(0%)	6 (22%)	8 (30%)	13 (48%)
Attitudes toward rural medical practice					
My rural residency experience has positively influenced my perceptions of what it would mean to practice in a rural setting	(0%)	2 (7%)	2 (7%)	19 (70%)	4 (15%)
My rural residency experience has negatively influenced my perceptions of what it would mean to practice in a rural setting	3 (11%)	18 (67%)	3 (11%)	2 (7%)	1 (4%)
My rural residency experience changed my mind about getting into rural practice post-residency	4 (15%)	9 (33%)	9 (33%)	5 (19%)	(0%)
I intend to live in a rural community once I complete my residency	1 (4%)	3 (11%)	3 (11%)	9 (33%)	11 (41%)
I have made plans to move or settle in a rural area after residency	1 (4%)	5 (19%)	6 (22%)	9 (33%)	6 (22%)
I intend to set up a rural practice once I complete my residency	2 (7%)	2 (7%)	8 (30%)	9 (33%)	6 (22%)
I have made plans to set up a rural practice post-residency	2 (7%)	9 (33%)	6 (22%)	5 (19%)	5 (19%)
I would recommend a rural residency placement for other students	0 (0%)	0 (0%)	0 (0%)	15 (56%)	12 (44%)

In terms of experiences during their training, rural residents provided positive ratings for the support of their preceptors (96%, *n* = 26) and other physicians and colleagues (93%, *n* = 25). They said they were provided with the appropriate level of responsibility (93%, *n* = 25) and patient load (85%, *n* = 23). The other amenities and attributes related to their training were mostly rated well, but residents provided lowest ratings for (i) adequate study rooms and resources, (ii) adequate recreation services, (iii) social connection to the community and (iv) adequate mental health resources. In addition, some physician trainees indicated feeling fatigued often (59%, *n* = 16), stressed often (44%, *n* = 12) and burnout often (37%, *n* = 10).

### 3.3 Perceived impact of COVID-19 pandemic

**Figure 6:**
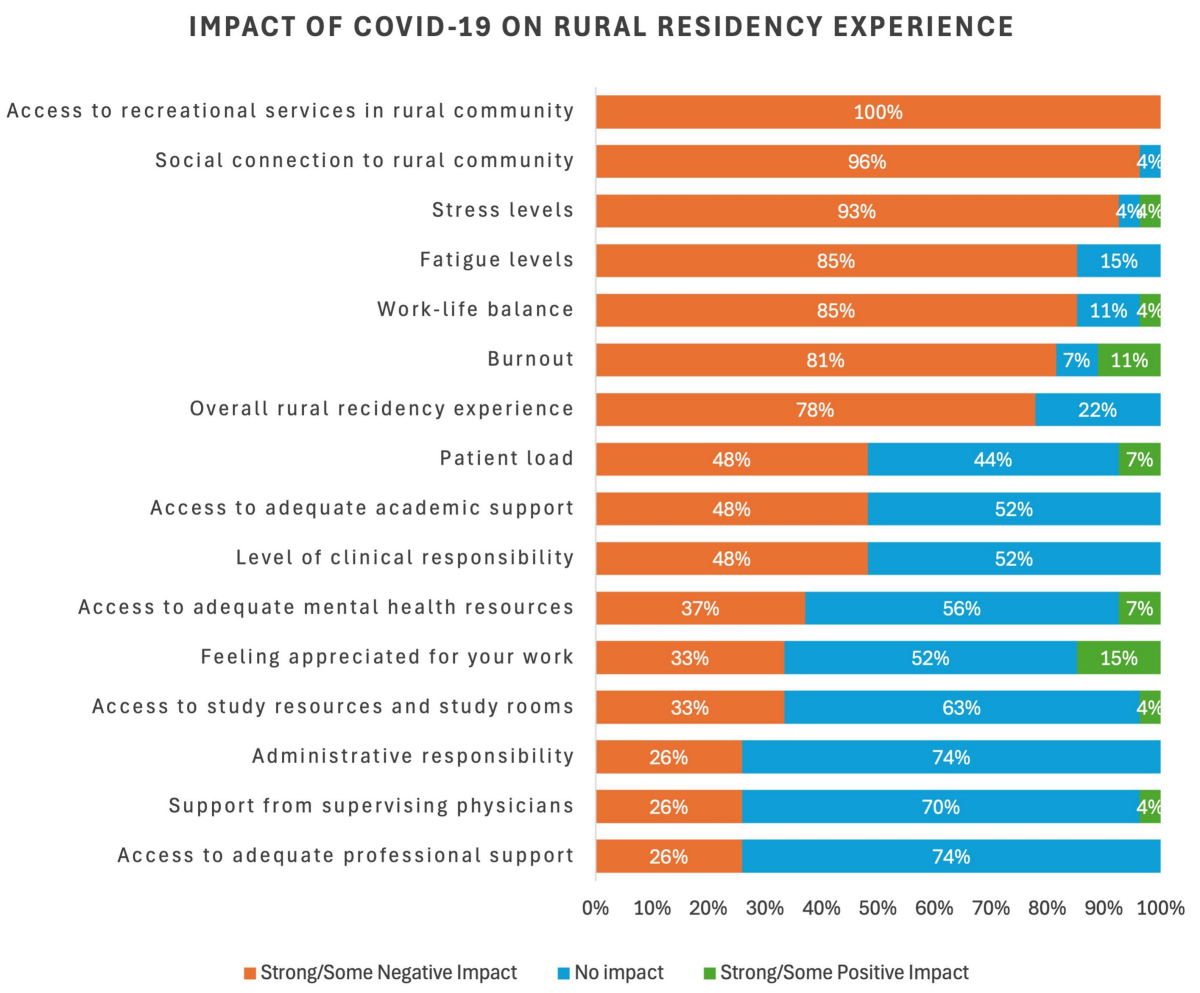
Perceived impact of COVID-19 on the rural residency experience.

The residents described impacts of the COVID-19 pandemic on their training (Figure 6). Some residents felt they were appreciated more for the work they did (15%, *n* = 4), while access to professional support, support of their preceptor and residents’ own level of administrative responsibility stayed the same. Additionally, an equal proportion of residents reported being positively or negatively impacted by COVID-19, in terms of (i) patient load, (ii) access to academic support and level of clinical responsibility.

On the other hand, most of the residents felt most impacted negatively in terms of:

- Access to recreational activities in the community (100%, *n* = 27)
- Social connections to the rural community (96%, *n* = 26)
- Stress levels (93%, *n* = 25)
- Fatigue levels (85%, *n* = 23)
- Work-life balance (85%, *n* = 23)
- Burnout (81%, *n* = 22)
- Overall rural residency experience (78%, *n* = 21)

### 3.4 Thematic analysis

#### 3.4.1 Theme 1: geographical isolation and resource-strained environments are challenges faced by rural family medicine residents

The residents described struggling due to separation from family, feelings of loneliness, exclusion, and cultural disconnection during rural placements, which were exacerbated by public health restrictions. They also had to deal with resource-strained settings such as housing challenges and staff shortages.

(i) The geographical isolation was a big concern for the residents, and the movement around it which impacted their schedule.

*R6: I was concerned more about the isolation, just because there aren't many residents in a rural setting. Whereas, if you're in the city, you're with a big cohort of co-residents, and there's going to be a lot of academic days where residents would be assigned a project and talk about it during academic days. … And so, I knew that it was going to be a lot of driving, and I know that Alberta winters can be quite tough.*

*R3: I think just the biggest struggle is moving around so frequently. I think if programs would make our schedules that we moved a little bit less. For example, if I stayed in Medicine Hat for six months at a time in first year, rather than six months spread out, that would probably made my stress levels better. And just also affordability because we were driving so much, so I spent a lot in gas and we have some of that mileage covered, but not everything.*

(ii) Residents also sometimes encountered housing challenges in their rural placement, especially during the pandemic.

*R3: they [the Rural Health Professions Action Plan (RhPAP) (* *41* *)] supply us our housing. It's been really hit and miss. Some of the places I've stayed in have been really, really nice and I've enjoyed staying there... But other times, they've been really horrible, extremely dirty, broken down in really bad parts of town.*

*P2: The initial accommodation that RhPAP gave them was not good. We actually had to move them to a different residence because it just wasn’t adequate. And without making it too squeamish, yeah, when you have mice running around your house, that is not the best environment to learn. And so they really upgraded it to a better accommodation.*

(iii) Geographical distance also contributed to professional isolation and cultural isolation, as it was harder for residents to connect with others or participate in social activities.

*R5: I would say I have felt isolated, particularly, in Peace River because … it was during the winter when the roads weren't very good. I was one of the few learners up there. It was I guess slightly professionally isolating in a way, not that I wasn't around a lot of other collegial doctors but there weren't a lot of people at my level that I was around.*

*R7: It would be nice to connect with more people of my own culture [South Asian]. I’m dealing with family stuff that only potentially another person of the same color would understand culturally how to navigate it.*

(iv) Residents shared the challenges of moving away from their partners and family, especially during the pandemic, and were worried about their loved ones’ safety.

*R7: My parents are in Edmonton and for me, I was just worried over the course of two years, if I'm gone any longer, God forbid something happens, they might need me to take care of them. So for me in my initial rank list was built out of obligation to family I would say, as opposed to training goals and for my own self.*

*P2: With some,… sometimes it’s even what I call family life … and I'll give an illustration. She’s a single mother here but her husband’s working in another place. Now she’s trying to integrate, you know, having a baby out here, breastfeeding it, doing her residency. Yeah? So some of those are what I call the life situations that they’re working through to become a physician and having those stressors.*

(v) Closures of community spaces not only meant there were limited options for places to eat and play sports and for entertainment but contributed to their social isolation.

*R7: Stettler was smaller. So I did find it a little hard to live there because there's just not much around and not much to do. So for me food is important, good food options, but there wasn't really anything besides a couple of restaurants. And entertainment, there wasn't really much.*

*R6: I guess the only thing that has been affected is that I like to play sports and team sports, which doesn't quite happen if you're in a rural setting when you're new to the place, and you don't have fellow colleagues that you can reach out to to play some sports. And it's also quite hard to join a sports team because, definitely, I looked around.*

(vi) The shutdown of public spaces also reduced the rural residents’ opportunities for community integration.

*R3: When the restaurants and stuff were shut down, that was a little more challenging because you wanted to see the community and do things. For example, in Medicine Hat, they have a ball team there and a rodeo and those types of events and they were canceled. It was just that not everything was open and you didn't get a chance to experience the community and the events the community would normally hold.*

*R1: Experience has been not what I want. … My goal had been to, like, put down roots and get involved in the community and it was almost impossible to do that with everything shut down and not having already those made those pre-existing relationships. … even like things that I would normally do, like go to a gym, just like that was even closed, so it was like working out in my basement. So it was like, wake up, go to work, come home, stay at home, and then, like, go out and do grocery shopping on the weekend, and then repeat.*

#### 3.4.2 Theme 2: disruptions in training and constrained structures of support impacted the residents’ wellbeing

Structural and social mechanisms, such as rural–urban cultural gaps, strained local health systems, and the professional “outsider” status of learners, shaped both normal and pandemic-era experiences of isolation. Rural trainees shared experiences of disrupted supervision, canceled rotations, switch to virtual teaching, and reduced educational support due to COVID-19. They described high workloads and emotional exhaustion.

(i) COVID-19 was a significant disruptor of medical education training, as public health restrictions meant cancelations of in-person teaching.

*R4: I just can't focus that long staring at a computer all day... I have my surgery rotation, a lot of surgeries and things were canceled. So I definitely missed out there, not necessarily just on OR time, but just general things as well. You just didn't get to do or see as much. So it made an impact.*

*R7: For example, we get two academic days every month and those initially pre-COVID were in person, and now everything is virtual. So we don't get to meet in person, aside on occasion from the lifesaving courses like advanced cardiac life support, and pediatric life support. That was nice because we got to see each other, but that was like six, seven months down the road. So, that's been a little bit hard, and right now, there's so much concern from the resident group that mental health is suffering. First year residents don't know second year residents and vice versa. We don't have that same level of connection and that's been really hard.*

(ii) For rural trainees, the pandemic-era changes such as suspension of social rituals (e.g., retreats, welcome BBQ events), conversion to virtual academic sessions limited peer presence, leading to reduced opportunities for professional bonding and social connection with fellow learners.

*R5: We have this retreat that we normally do in Hinton, near the beginning of residency, normally in August. The whole thing got canceled for us. We had some weird Zoom thing instead, which was obviously not as fun as going hiking in the mountains.*

*R4: …to be honest, I don't feel super connected to my co-residents. I really don't, because we don't ever really get to do much together. We don't see each other on academic days. We're on Zoom, but it's just not the same.*

(iii) The residents noted that things became better when the pandemic restrictions for social gatherings were eased after COVID-19 vaccines became available.

*R5: All of our academics became virtual. We didn't get to see each other at all; we didn't get to socialize. I just felt like I didn't know them as well. Then now in second year, basically, since everyone has been vaccinated, things have really gotten a lot better and we've done a lot more together. We did our first course in-person. Two weeks ago or so? It was honestly the best thing ever. We spent three days where we would do our course stuff during the day and then we would go out for supper and hang out in the evening. It was really, really awesome. COVID really put a huge damper on things for sure.*

(iv) Training pressures were amplified by pandemic disruptions (e.g., canceled rural electives and accreditation demands).

*R6: Throughout first-year residency, we had so many courses being canceled on us. And it was quite frustrating, trying to find a place to stay to attend the course, and then trying to make the time to travel there, and then it being canceled very close to the event. But there are things that had to be completed, like courses had to be done. But they kept on changing. And I wasn't able to even know, like, "When was this course going to be happening? When should I prepare the material for this course? What should I study for the material for this course?"*

*R2: I had to cancel an elective because my preceptor ended up getting COVID... the other residents weren’t able to do their anesthesia rotation. We normally would be getting the EDE course for ultrasound; we can’t do that. The second-years, I feel really bad for them, actually, because they’re probably going to graduate without it, which is really important for a rural family physician to have.*

(v) Although it was rare, some residents reported a perceived professional “outsider” status of learners

*R3: I guess, maybe a family medicine resident in the city would have way less experiences and they are generally working with a goal to just be in clinic, so they may be less interested and put less work in. Whereas in a rural site, we're just expected to do anything and everything and so when you move from a rural site to an urban site, it feels a little bit demeaning sometimes because preceptors won't always trust your opinion or judgment. It's always like very helicoptering. They're always watching everything you're doing, so there's very little independence. And then I would say at rural sites, the goal usually is for the learners to actually be learning because at rural sites, they generally don't have residents.*

(vi) Rural residents described the toll of high workload and long clinical shifts, especially in the internal medicine/ICU rotations. The residents reported difficulty in maintaining a good work-life balance and felt burnt out. Some rotations were more stressful, and some residents reported that once they moved to rural Family Medicine, they were able to recover their footing and everything was fine (R5).

*R7: At Brooks, I was too tired to integrate and do anything else because the shifts were 12 hours every day. So I don't think I got as much integration, although they were really cognizant of providing a really nice, welcome package and saying these are the resources in the community, and they offered a lot.*

*R2: I think that it depends on the rotation you’re on. For instance, surgery and obstetrics tend to be pretty heavy because they do 24-hour call …. it tends to be a bit intense, and it burns you out a bit… it’s definitely hard on you, mentally. Especially the rotations where, like, you’re not sleeping properly because of the 24-hour shifts. … it’s not healthy for anyone, ever. I don’t know why the hell we still have to do that, to be honest. It’s like, should be illegal.*

*R5: It does really depend on the rotation. Like I was saying, OB-GYN and internal and gen surgery were the three where I was like, "Oof, I'm burning out" or my anxiety is fluttering or something like that. When I was on my internal medicine/ICU, because it's combined for us, I was this close to taking a leave for my mental health, but my breaking point was right near the end, so I pushed through a few... It was like a week or something left. Internal medicine is also busy and ICU, in particular, was quite taxing. It's just that the mental bandwidth that was required to get through the day was so much higher, so even if you're seeing the same amount of patients, it was a lot more emotionally taxing.*

(vii) Some residents grappled with norms around physician toughness, self-reliance, and stigma around mental health.

*R4: There was one night where I got sent to the NICU for a baby that was not doing well and it was way over my head. I was stressed. I'm not a stressed kind of person, that's not really my personality. That night was like stress and anxiety that I don't usually experience. So that was one of the first times in residency that I was like I am in over my head here. I need to be bailed out right now.*

*R2: I was extremely depressed, and I really didn’t know that I was. And she kind of said to me, … I think that you should consider taking some time off, and I really did not want to hear that, because I don’t want to be off cycle. I want to graduate with everybody. That was my thing that I kept focusing on. And I was always trying to kind of be like, well, what if I just take two weeks off, and I can use my vacation, or I can, work extra just to make sure that I don’t have to take much time. And she was kind of like, … That’s really not a great idea. Like, this is kind of what I’m concerned about with you, this is what I think you should do. And, in my head I was like, I can handle this because I’ve pushed through for so long. … And the second I decided to take time off, it was, like, the craziest thing because it was like everything just came crashing down.*

(viii) Institutional responses to the pandemic-altered learning environments such as adjusted program processes and scheduling caused confusion and stress for the rural residents.

*R2: It is stressful to be switching from rotation to rotation; you never know who you’re working with. There’s always uncertainty. You’re always being evaluated all the time. You never really actually know what the hell you’re doing. The thing about our program is that I find a lot of people struggle with, and I think it does add a lot of stress, just, and I don’t know how else you would do this, but we don’t get, like, a schedule way beforehand. We don’t have, like, set hours. It’s very dependent on what comes in and who you’re working with, which you might not even know until the day of.*

(ix) Some residents commented on the structural rural disconnection such as urban-centered policies or processes that did not fit rural contexts.

*R7: I'm struggling with it every day honestly. I think supports and resources, are limited for a rural centre. You often have really difficult patient encounters and sometimes you just want somebody to talk to, and vent, and complain to a little bit and just share what happened. And that's hard when somebody is not right there, because otherwise then you have to make separate time, call them, et cetera. It's helpful if somebody's in proximity, I think the urban program is a little better in that regard because at least you have more people around, but rurally it's very hard to have that connection. I think we brought this up with PGME and UC to get supportive resources, but … the best they can really offer us is skip the dishes gift card for you to have a Zoom meeting over Christmas holidays for example, have a virtual holiday day dinner.*

*P1: So as far as formal [wellness supports], I would have to divert to whatever the programs are doing. It wasn’t part of my purview on site.*

(x) Residents noted the difference between settling in a rural area and establishing a rural practice. They perceived limited institutional supports for rural communities, especially for resources for transition to practice after residency.

*R6: In terms of transitioning to practice... It's not a structured course that teaches you how to find a job and how to get started. We don't have a natural lecture or a course on a list of things that you need to do, and how to find a place to work, and how to set up your own practice. And so, now I’m halfway in my second year. And after training, now I'm reaching out to my preceptors and asking them, "Look, can I join you guys in the future to locum, or is any particular place that you would advise me to go to do the initial startup to transition to practice and to get more experience?”*

*R4: Settling, for sure. Establishing my own practice, probably not. I'd rather join someone's practice and that's just because of some of the issues and stuff I've seen from my preceptors. They recommend joining with someone if you can when you're starting out, because your first year out of residency in a lot of ways is actually your biggest year of learning. … Just the business side aspect of running your own practice is an additional stress and there's no real teaching or guidebook to it.*

#### 3.4.3 Theme 3: effective mentorship, community integration and role identification fostered adaptive resilience

Community preceptors have a foundational role in training residents, by providing effective mentorship, as well as both practical and emotional support, which fostered residents’ learning and personal growth. Residents reported inner growth, perseverance, role identification and meaning making despite adversity. Furthermore, getting to know and being integrated into the community promoted a sense of belonging for the residents.

(i) Preceptors supported resident well-being by establishing clear expectations, fostering trust and open communication and making adaptations to accommodate different learner needs.

*R5: In Spirit River, I felt very supported. He checks in with me every day, even if I don't ask questions, even if he doesn't see me to be like, "Hey, are you doing okay? Have any questions about your clinic or any concerns about the inpatients?" He's always available for me. Then also he has called me in if there's something interesting that I could learn from. For example, a patient came in with a dislocated shoulder and it's not all the time that you actually get the opportunity to [do] something like that.*

*P2: Now, if the stress is coming from workloads, yeah, you tend to adjust it to see if you can accommodate so they’re more comfortable in a stress level. And the complexity of the patient varies according to the amount of stress they have. For some of them, like, I really like them to take care of the patients, right? But if they’re too complex for them, you start giving them easier patients so they’re working more in their comfort zone. The other thing, and just going a little bit more broadly, some are very good in certain areas and not good in other areas, and you still would move into the areas where they’re not that good because they still have to have that comprehensive education. It’s always easy to do what’s easy.*

(ii) Effective preceptors strike a balance of providing both practical support and encouragement and allowing residents to take *on* progressive responsibility.

*P2: And I’ll comment that the first-year residents, … when they transition from being a student to being a resident, you really try and encourage them into that transition – that now they are physicians, you know, they have the ability to treat patients versus a student that has to report every time they see a patient, right? And I give the comments. I really enjoy the first couple of months when they start. I also enjoy the last two months of the second year when they’re going, when you really try and develop them to the stage you are not going to be out there; you are going to be on your own. And in your practice here, we are going to let you do that. We still go over the patients, still supervise them, but really you take care of them as you think you want to do when you’re going to be out on your own or in the next month or two months.*

(iii) Residents considered social relationships are the most critical for mental and physical wellness and staying connected to family and friends was most important and a “huge thing for my wellness” (R5), even if it meant “just talking by phone to my partner and friends and family” (R3). Residents also learned to lean on their fellow leaners to cope.

*R5: In the resident group, we have different roles, different people do different things to help self-manage and one of those roles is we have a social rep for each year. They often plan stuff for us to get together. Even beyond our social rep, we have a couple people that just really love to host. They have people over quite often. We've done games nights and wine tastings, paint balling. … We go for hikes and walk dogs together and get coffee and that sort of stuff.*

*R6: So, because we're such a smaller cohort of residents, we're all in touch with each other. We're on apps. We're on WhatsApp. We're on Messenger. We're always in touch with each other. And so, I personally haven't reached out to any resources directly, but I definitely spoke to my fellow colleagues. And also, as an R1, I was able to speak to residents in the year above me. So I think the support that I have mainly got was from other residents.*

(iv) Rural residents found ways to overcome geographical barriers and maintain social connections, which includes the use of modern technology.

*R5: Within the resident group, we have a Slack group with different channels in it. The stuff that we talk about could range anywhere from what was that requirement that we have to do? When is that due? To does anyone know a cellphone number or a good elective that I can do next month to today I really screwed up and I feel like a failure and then everyone else can reassure you and lift you up to sharing funny TikToks. It's a good community to have when people are spaced out and aren't necessarily seeing each other all the time.*

*R7: Speaking to that, I guess one thing we have been able to implement though, because this problem came out last year … was residents were saying, there's a lack of connection, when do we talk to each other? When do we discuss difficult cases? When do we interact? And so we were actually able to implement... We called them wellness sessions at first, but now we call them reflections in medicine, which is basically Zoom sessions…. And basically they're designed for us to get together with our co-residents virtually and talk about mistakes we've made in medicine or difficult patient encounters. And people have found that to be better in terms of having the connection with their colleagues, myself included. I've felt better about having them.*

(v) Staying physical active, being with nature or having a pet helped residents to manage their stress levels.

*R6: I like to stay physically active. That has really helped with my both physical and mental wellness. I really find that physical exercise helps me to relax and stay focused. I found ways to maintain my physical activity just because I carry with me simple exercise equipment. I would either exercise at home or go to a nearby gym in the community. … Like, I brought roller blades and a skateboard, and I brought simple exercise equipment with me. And then I even bought a free-standing punching bag to just practice kick boxing on. So simple exercise equipment that was not too heavy and that was able to fit into my car. I carried them around all the time. And so, that was a big part of my mental and physical wellness.*

*R3: I started running, which I found getting out and getting more exercise was really helpful, just a way to burn off energy. Then I did... I think U of C offered some online counseling, which I did for a brief period of time, but didn't really find it helpful. It's not really my thing.*

*R5: I tried to get outside when I could motivate myself to go out in the cold. I did some walking because there's a big ski hill that's really nice for hiking, walking, whatever you want to call it. … I also have two pets at home that I have gotten during the pandemic. I have a cat and a dog at home, who give me a lot of mental health support.*

(vi) Residents said that exploring and getting involved in their community and making use of available community recreational spaces helped them to destress.

*R5: Then just doing things that are relaxing and fun for me. My husband and I took up, is a bit strong, but took up squash a little bit at the local rec center... Oh, I forgot that part. Our program gives us free passes to the rec center. That's obviously a wellness thing. I did not remember that before. Yeah. We get a free annual pass to the East Link Center. Using that, we went and played squash.*

*R6: I really enjoy actually driving on the highway, looking at the sceneries, and kind of exploring a new place. I found that to be quite relaxing… But I overall enjoyed, actually, roaming all over Southern Alberta, and meeting new people, and seeing new places.*

(vii) Moreover, residents valued being welcomed to the community through their integration and retention initiatives.

*R5: The people at the town office were very welcoming and they entered all the local docs into a draw as part of their retention committee, so I won a prize this week. One of my patients invited me to a block party in town for Christmas, which I did not attend but I feel like that's still an aspect of community integration.*

*R4: They were so excited always to have a hometown doctor back where they're from. So I guess as far as reintegrating goes, I've been welcomed very, very well, not like from staff. They're almost motivated more to teach you even better because they know you'll be one of their colleagues, … it's very appreciated from me because I'm the one reaping the benefits. And the patients as well, it's awesome when they're just so happy to have you around. They're a bit more understanding that you're new and patient with you.*

*R7: And I think they gave us a free pass to their local leisure center, which was really good. I think I've spent time with my colleagues outside of work, as the pandemic permitted in between waves anyway. And even preceptors would invite me out to dinner and spend some time and that's been really nice too.*

(viii) Reflective practice helped residents to make meaning and gain a sense of purpose to handle the day-to-day stresses of rural medical training.

*R3: Family Medicine was something I decided to go into last minute. I was a little bit anxious about that decision and wasn't used to being away from home. So at the beginning of my residency, I think I really struggled with some depression and a bit of anxiety as well. But since then, I've stabilized out and think things are about where they were or even less stressful compared to medical school.*

*R5: In first year, we do four months in a smaller community... It's pretty well outfitted. It's completely run by family doctors but a lot of them have extra training, so they have at least two GP anesthetists, at least two GP surgeons, and then a lot of docs that do obstetrics and hospitalist stuff. Everyone does emerg and everyone does clinic. It was pretty much the biggest scope I had ever seen from a family doctor perspective and then I was also given quite a bit of independence. Like in a safe, supported space but a lot of independence, even as a first-year resident to see my own patients and have my own schedule. I really, really liked it.*

*R6: Obviously there's stress and the frustration of doing the residency during COVID. But I will say it's nothing that's out of control. I am still able to manage everything and still focus on my learning, and I still got to have a decent work-life balance. So I feel like it added more stress, but it didn't throw off my wellbeing. So I don't think my wellbeing has drastically decreased. It's just the additional stress that was still manageable.*

(ix) Residents noted role identification, moral commitment and perseverance helped foster adaptive resilience.

*R5: After that rotation [in Peace River], I was even more convinced that rural family med was what I wanted to do. … Then in my second year, there was just that I guess moral distress is the best way to put it, that these are people that are in the same profession as me where it's like do no harm and try to protect patients and then they're going against everything that evidence has suggested is the right thing to do. Mental health-wise, I think is the biggest impact it's had on my residency because along with the regular stresses of residency, you have to deal with this distress of trying to be the role model, follow guidelines when the government has been quite inconsistent with restrictions and at the same time, trying to keep our own head above water and then people asking you for vaccine exemptions and mask exemptions and having to repeat the same spiel over and over and having it not change anyone's mind and burnout from other healthcare professionals.*

*R4: I still feel like in order to be my best doctor self, a rural practice is going to provide that because it allows me to be the sole care provider, in a lot of ways, to be very well connected to my patients and my other medical colleagues around me, because resources are limited. I think there's a good team dynamic, and so I like that. So I think in a rural location you do get to maybe know some of your colleagues a bit better, because there's not as many of you, and so it's nice to work with them in that way. So that's a motivation, as well, to stay.*

(x) Residents appreciated the institutional flexibility and responsiveness.

*R5: I love my program for this. I personally haven't had to take any sort of leave or anything but other residents in the program have for different reasons. Some examples, one found out that her mom had pancreatic cancer and very easily was able to take time off in order to support her mom and then was reintegrated later. Another one was in a car accident and had an awful post-concussion syndrome and took time off and then was gradually reintegrated instead of being thrown in right away. Lots of people take mat leave with no issues.*

*R7: I think the only support I've seen that needs to be offered..., we should have the flexibility to take that time off without us being penalized for it, because that ultimately helps you with your wellness. If you're dealing with something major, you want to have that flexibility and time to deal with it and not have to worry about your day job.*

*P2: Well, and the most dramatic was, and I don’t think it can be any more traumatic than this, one of them lost his brother suddenly from a heart attack, I mean, out of the blue. Totally unexpected. And then to help him through some of the grief. And I actually requested for him that he gets the time off, even talking to [Dr. RS] who’s a director, saying that this is what happened. I think this is what we did. Then after the two weeks off, then accommodate him as he comes back to work, to make sure he’s not – that he can concentrate, that he can learn and get back into the routine. Now, he was – I could call him one of the tougher residents that he came back and was able to concentrate and that sort of thing, eh?*

(xi) Yet, some residents spoke about the lack of knowledge of formal wellness and wellbeing supports.

*R5: I don't know if there are a lot of formal program-specific supports. I know there have been times where I have struggled a bit in residency and one of the supports that I've utilized is the physician and family support program. I guess for residents who don't have a family doctor, there is a doctor for the residents, if that makes sense, while we're in residency. That would be a resource that could help too. Otherwise, I don't know of any formal supports.*

*R7: Other supports, unfortunately I think it's a little limited. But I don't know if that's a rural specific thing, I think a lot of people are seeing that even in urban centers too. I think it's just the nature of the pandemic. They constantly send us these emails like, oh, there's the physician family support line from the AMA and whatnot. But I mean, I don't know. It can only take you so far. I think it just depends how each person defines what being well to them is and what they want.*

(xii) Most residents viewed the residency program positively and have indicated the willingness to stay and practice in a rural community.

*R5: Rural programs are just so much more reasonable and it feels like there's less red tape because there are fewer people that they have to manage. People just know you on a personal level and it's so much better. You have say in your schedule, you can move your call around, you have a ton of say. You don't have to take your vacation all in one block, you can space it out, you can take one day off here and there if you have a special, I don't know, hockey game that you want to go to once a week. Yes, rural residency is the best. I think the only way to train but, obviously, I am very biased. My rural residency has been everything I've expected of it and more. And because I am interested in doing some emergency work, I'm actually very happy with the calls I'm doing*.

*R6: I know that medicine in a rural setting is excellent. I really like the work in rural family medicine. But the only thing I'm trying to think and debate on is will the lifestyle, and is the rural setting supportive for starting a family? I think that is a factor that I still need to consider, and which is why I haven't fully decided on doing a rural setting yet. But ultimately, if I were to settle down in a place, will I be able to find a partner? Will my partner be able to find a job in a rural setting? And is the rural setting going to be a good place if I decide to have children in the future? And will this be a good place for them to go to school and to learn?*

(xiii) Rural residents have developed a rural professional identity rooted in service, in spite having concerns about certain government policies.

*R6: The only thing about that really concerned me was Bill 21, where their idea was to restrict where we would work in a rural setting. Would I end up being stuck in a real setting, and don't have the option to move to other towns or even to move back to the city if I wanted to?*

*R4: I want to be a good doctor. That's been my top priority, regardless of what the government is saying or issuing. So I still felt rural was the best way I could do that. … So, rain or shine, whatever the government says or does, or doesn't do, I've already made up my mind that I'm just going to stick it out. So I haven't let it impact me too much. Does it concern me when I hear about stuff? Yeah. Do I think physicians need to continue to make efforts to cause changes and advocate for themselves? Yes. Do I support that? Yes. Will I? Yes. It's not like I'm just totally turning a blind eye to it, but I'm still focusing on becoming a good physician before everything else.*

Additionally, residents coming from a rural background would like to practice also in a rural community.

*R5: The big thing is I have roots in rural Alberta, I know how to live the [rural] lifestyle, I have a huge support system in this area and I just know how the culture, especially rural northern Alberta. Then from a medical standpoint, I just love my rural rotations, I love medicine rurally.*

### 3.5 Qualitative interpretation

#### 3.5.1 Mapping of themes and sub-themes to Bhaskar’s laminated systems model

**Figure 7:**
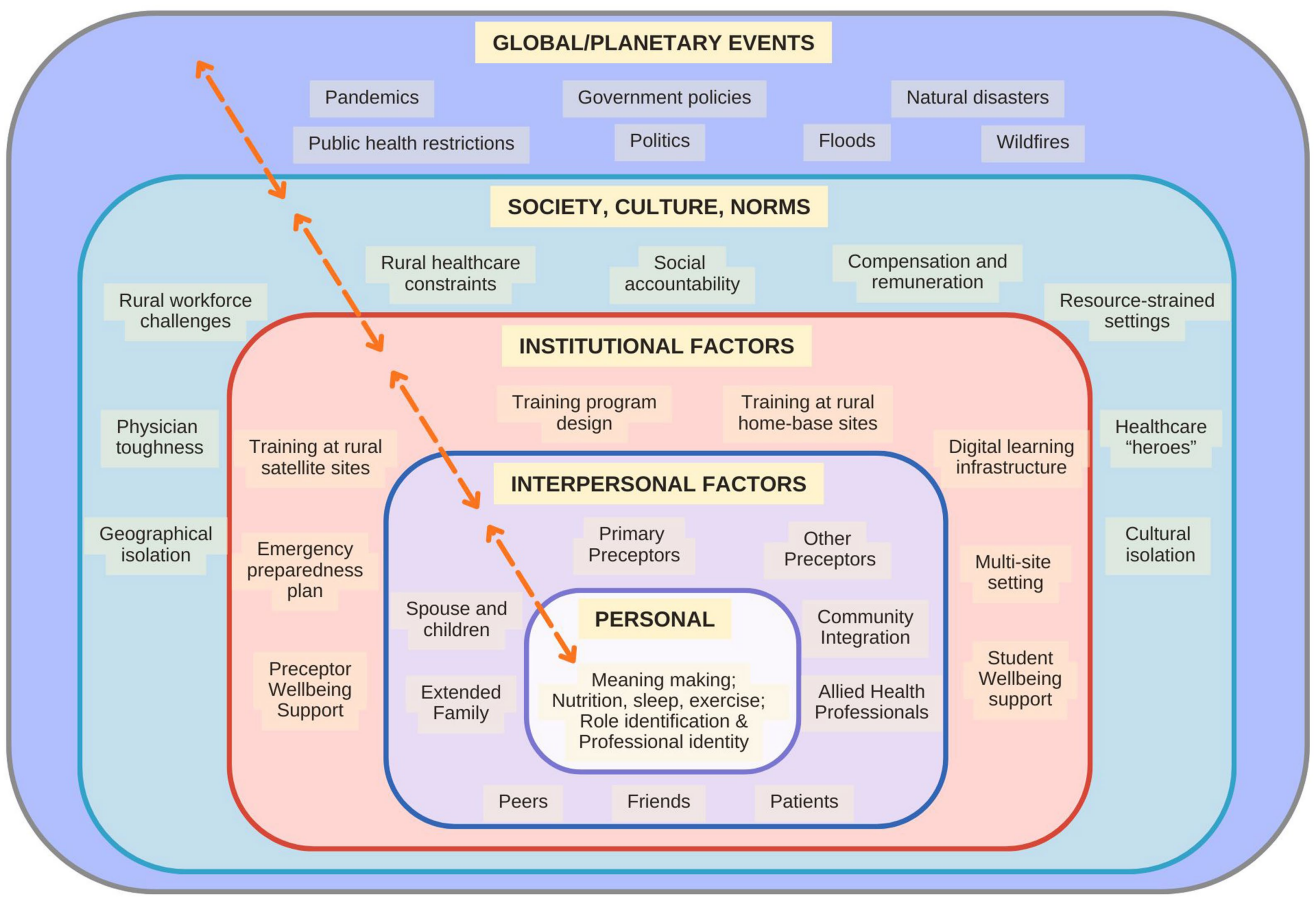
Concept theme map to Bhaskar’s laminated systems model.

The authors conceptualized the wellbeing of rural family medicine residents as shaped by interactions across several layers within Bhaskar’s LSM, with mechanisms at different levels influencing the other levels (Table 3). These are represented in Figure 7, adding arrows as directional symbols to help readers appreciate the dynamic (and individual) nature of how the various factors of the training environment can support or detract a resident’s wellbeing.

**Table 3:** Mapping of themes/sub-themes to the Bhaskar’s laminated systems model.

LSM level	Codes (sub-themes)	Example
Personal (Physical/psychological level)	Sleep, diet and nutrition, exercise and emotional resilience. Cognitive appraisals of stress, anxiety meaning-making processes.	A resident experiencing insomnia due to workload and personal stress illustrates the interplay between physiological strain and psychological processing
Relational/interpersonal (Micro level)	Relationships with peers, preceptors, other staff, patients, and community members. Quality and availability of mentorship or social support.	During COVID-19, diminished in-person contact with colleagues and mentors led to feelings of professional uncertainty and isolation.
Institutional/organizational (Meso level)	Training program design, faculty availability, digital learning infrastructure. Institutional response to emergencies like the pandemic.	A site lacking reliable internet hindered access to online modules, showing how technological gaps at the institutional level influenced individual wellbeing.
Cultural and societal (Macro/Mega level)	Norms around physician toughness, rural values of self-reliance, and stigma around mental health. Broader discourses around healthcare “heroes” and expectations for resilience emergencies like the pandemic.	Residents may hesitate to seek help due to internalized cultural expectations to “power through or soldier on,” shaped by both medical culture and social stigma (“cultural silence”).
Global/planetary level	Closure of community spaces during pandemics and other public health emergencies. Policies for disruptive events (e.g., pandemic health policy), health resources allocation.	Health policies, pandemic restrictions, increased clinical workload and fear of virus transmission impacted individual wellbeing.

#### 3.5.2 C–M-O configurations aligned with Bhaskar’s laminated systems

Table 4 illustrates how C-M-O configurations operate across LSM’s layers, showing the relational *dynamics between levels and how outcomes are complex and emergent rather than linear, which* informed the refined program theory (Figure 7). These configurations reflect broader institutional resource inequity in rural medical training.

**Table 4:** C-M-O table aligned with Bhaskar’s laminated systems model.

LSM level	Context (C)	Mechanism (M)	Outcome (O)
Personal	Nutrition and sleep, physical and mental health	Coping styles, emotional regulation, sources of support (e.g., family or spouse)	Resilience or exhaustion
Personal	Support for work-life balance	Adequate housing, lack of community recreational resources	Stress, fatigue, burnout, reduced sense of value
Interpersonal	Strong resident-preceptor relationships	Mentorship, informal emotional support (i.e., feeling supported and understood)	Increased resilience and engagement
Interpersonal	Strong rural community relationships, mentorship	Interpersonal trust and rural identity	Improved coping, emotional support
Interpersonal	Rural community integration	Empowerment through recognition; identification with rural community and role, sense of purpose	Greater professional confidence and purpose; renewed commitment to rural medicine, moral fulfillment
Institutional	Training at rural home-base sites	Established community; availability of supports	Meaningful engagement in wellness and mitigation strategies
Institutional	Training at rural satellite sites	Increased isolation; lack of formal supports	Decreased perceived wellbeing; resident burnout; perceived invisibility
Institutional	Disrupted training pathways (e.g., virtual academic sessions)	Anxiety over competence, disrupted identity formation	Loss of confidence; stalled learning trajectory
Institutional	Responsiveness, centralized support (or lack of), program delivery changes	Inconsistent policy adaptation to rural realities (or lack of), bureaucratic disengagement	Feelings of invisibility, abandonment or neglect, poor training experiences
Cultural/Societal	Shortage of rural workforce and clinical resources	Inadequate supervisory continuity due to staff shortage	Resident stress, reduced confidence
Cultural/Societal	Structural rural healthcare constraints (i.e., resource-strained setting)	Persistent inequities in access, resources and support	Long-term fatigue, moral injury; anxiety about future practice
Cultural/Societal	Norms around stoicism, social accountability	Internalization of professional expectations duty to serve in rural medicine	Profession identity affirmation or guilt/shame
Global/Planetary	Disruptive events (e.g., pandemics, floods, wildfires, etc.) in rural settings	Reduced supervision; increased isolation; increased responsibility	Lack of community, reduced access to education, burnout, decreased wellbeing
Global/Planetary	Policies for disruptive events (e.g., pandemic health policy), health resources allocation	Policy mismatch with rural realities; structural inequities, rural marginalization	Disconnection, strained services, reduced sense of value, stress

#### 3.5.3 Integration of quantitative and qualitative findings

Qualitative insights are further triangulated by the findings from the quantitative survey data. While many residents reported positive experiences in their rural residency and felt well-supported by their programs, wellness concerns around burnout, stress, and isolation during rural training were evident. Majority (96%) of the residents reported a highly positive perception of the professional life of a rural physician, almost all had looked forward to their rural training. In terms of experiences during their training, almost all rural residents provided positive ratings for the support of their preceptors and other physicians and colleagues. They felt they had an appropriate level of responsibility and patient load. On the other hand, perceptions of satisfaction were rated much lower for adequate study rooms and resources, adequate recreation services, social connection to the community, and in person mental health resources. Nearly eight in 10 residents reported a negative impact of the pandemic crisis on their overall residency experience. They universally noted that the closure of community recreational spaces had the greatest negative impact, which diminished opportunities for physical fitness activities, mental decompression, and community integration. This was followed by disruptions of social connections within the community, elevated stress levels, feelings of fatigue and exhaustion, and burnout.

## 4 Discussion

This mixed methods research study examined the experiences of rural program Family Medicine (FM) residents during their training using a critical realist approach that incorporated Bhaskar’s laminated systems model (LSM) (27–29). The authors sought to explore and describe the mechanisms that influence FM residents’ wellness and wellbeing during rural medical training. Ranatunga and Pagliano posited that wellbeing is a complex phenomenon and to understand the complexities of the underlying factors, the explanations may not be reduced to a single level (30). In pursuit of robust explanation, Byrne proposed the use of Bhaskar’s process model to map the impact of different levels of factors and changes in the environment of the individual person (31).

Application of the LSM revealed how wellbeing is influenced not just by experiences, but it is shaped by multi-layered social, structural, and personal mechanisms across time. These mechanisms (both stressors and support structures) resulted in a refined program theory. The authors found that rural physician trainees navigate unique challenges due to geographic distance, strained resources, and increased workload.

The authors uncovered that wellbeing among rural residents is deeply shaped by structural constraints and social dynamics that extend well beyond individual experiences. At the interpersonal and institutional levels, residents reported feelings of social and professional isolation. During the global crisis, residents felt the separation from their friends and families more acutely due to concerns of infection transmission. Urban-centered decisions by the institution, resource allocation, and one-size-fits-all educational directives also created additional tangible disparities for rural learners. These feelings were compounded by structural deficits such as limited community spaces, housing challenges, and fewer peer interactions, that reduced opportunities for reflective dialog and support. In critical realist terms, the context of rurality, combined with mechanisms such as limited institutional investment and professional solitude, produced outcomes like emotional strain and disconnection. The mechanism of geographic and professional isolation was activated in the context of under-resourced rural health systems, resulting in outcomes that negatively impacted wellbeing and learning trajectories. These experiences align with LSM’s focus on how societal and global systems (such as healthcare infrastructure, health policy) could penetrate the personal domain.

During disruptive events such as the COVID-19 pandemic, both residents and preceptors described breakdowns in continuity of education, formal learning structures and vital peer interactions. These disruptions occurred at multiple levels of the LSM. The pressures of training were amplified by pandemic disruptions (such as cancelations of academic days and operating room experiences). Residents reported being frustrated and stressed with cancelations of courses and electives and the short notice and last-minute changes in their academic schedules. They described the mental and physical toll of the clinical workload, especially the 24-h on-call shifts that left them sleep-deprived and fatigued. This made it difficult to maintain a good work-life balance, leading to burnout.

At the institutional level, dependence on limited rural staff and faculty, program suspensions and policy changes undermined curricular stability. The pandemic-altered learning environments and the suspension of in-person academics tremendously reduced peer-to-peer interactions and failed to foster a sense of camaraderie and support among residents, stalling the residents’ learning trajectory. Yet, the type of rural location appeared to moderate resident wellbeing in this context, and improvements to formal and informal resources available at rural satellite sites could improve resident experiences.

At the societal level, public health mandates led to closure of community spaces such as libraries, restaurants, and recreational centers. These mechanisms affected the residents at the individual level, causing psychological stress, uncertainty, and diminished motivation. These disruptions demonstrate how institutional and global events activate mechanisms such as contraction of institutional support, leading to outcomes such as increased stress, burnout, disorientation and reduced education satisfaction among rural FM residents. This reinforces that value of viewing wellbeing not as a static outcome but as emergent property shaped by many layered contexts and mechanisms.

Many residents described role identification rooted in service, finding purpose in rural care, and drawing strength from family supports and community embeddedness. Here, the context of a supportive rural community, combined with other mechanisms like relational continuity and professional role modeling, facilitated outcomes of strengthened identity, resilience, and meaning making. The authors’ findings support Ranatunga and Pagliano in that explanations of wellbeing may not be reduced to a single level (30). Like Byrne proposed the use of Bhaskar’s process model to map the impact of different levels of factors and changes in the environment of the individual person (31), the authors similarly found the interweaving of various personal, cultural and societal as well as global factors associated with residents’ wellbeing clearly illustrates the necessity of adopting an ecological approach to wellness and wellbeing promotion among residents (42). Connectedness has been shown to foster resilience in medical education (43). Similarly, community integration has been linked to professional identity development and subsequent rural practice intention, and hence, serves as an important resilience mechanism that is enabled by small community networks (44). Previous work also posited that professional identity formation works as wellbeing mechanism by nurturing connectedness and relationality (43, 45), and it is vital in training adaptive and compassionate physicians (46).

In addition, community preceptors are a particularly vital mechanism of wellbeing among residents and are integral in the training and development of rural physician trainees. Residency programs rely on their effective mentorship in establishing clear expectations, fostering a sense of community, role modeling and providing both practical and emotional support to the residents. However, preceptors may not be able to fully participate as a wellness resource for learners due to lack of awareness of formal processes. The lack of knowledge on available formal program supports may diminish their effectiveness as mentors and sources of support for learners.

Academic medical institutions must continue working on initiatives such as decentralization policies that support the training of rural physician trainees. Factors that appear to contribute to success include nurturing learning environments and the availability of mentorship. However, attempts to improve residents’ wellbeing would be inadequate without paying attention to other social, systemic and structural healthcare factors such as staff shortages in rural settings (45).

## 5 Implications

The findings allow medical educators to recognize the systemic, structural, and relational conditions that promote resident wellness and support resilience. This could potentially encourage the design of interventions that are attuned to the laminated nature of reality, targeting not only individual coping strategies (e.g., self-care) but more importantly structural reforms, institutional continuity, and relational support systems.

The study highlights the importance of supporting preceptors and trainees during residency and addressing resident perspectives on post-residency transitions to independent practices. The sustainability of rural residency programs depends on recognizing and strengthening key supports including designated trained mentors, access to formal wellness resources, safe and stable housing, flexibility with scheduling, and limiting turnover between geographic locations (23, 24). Examples of institutional supports can guide effective mentorship and peer support for mentors. Readily accessible wellness resources at each placement site, free passes to the community recreational centers, athletic parks and programming, and an emergency preparedness plan with a dedicated point of contact could benefit trainees. Regular assessment using validated tools, such as wellbeing measures can help identify programs or sites requiring additional support. Furthermore, disentangling wellness from wellbeing warrants further examination in training contexts such as rural family medicine (47). These efforts are critical, as residents who feel supported and integrated into rural communities are more likely to remain in practice, and physician wellbeing is closely linked to patient safety and quality of care (45). Future research should also examine the wellness experiences of urban residents undertaking rural placements, as differences in training culture, isolation, and community integration may expose significant gaps in available supports for this group.

Rural medical education settings do not have the size or scale to support the same array of resident wellness resources deployed in urban settings. While local wellness supports are crucial for day-to-day support, specialized resources may be required by some residents at some times. The needs of individual residents, and the communities they will be working in when need arises is unpredictable, so wellness support systems must address both routine wellness supports as well as access to escalating resources when required.

Local infrastructure to support wellness is a key element of sustaining wellness during residency. Ensuring that preceptors are aware of wellness resources, and offering faculty development opportunities to empower preceptors to address resident wellness as a routine part of education is critical. Ensuring that housing and living arrangements for residents are suitable is also an important basic component of wellness support. Engaging the residents beyond the clinical environment and their living arrangements further supports basic wellness. Organized engagement with rural municipalities to ensure that residents are aware of professional and recreational opportunities in a rural community can support a successful experience.

Access to centralized resources must also be considered, and since there are always residents working in rural communities at a distance from the centralized campus, consideration should be given for how all wellness services are delivered at a distance. Access to counseling, including in person counseling and virtual options, is important, especially when a resident identifies a need for enhanced support. Opportunities to connect with peers is particularly important and ensuring that time is protected for academic days, when all rural residents convene together, must be a priority. Regularly scheduled meetings with mentors, counselors, or rural program leaders can also offer an opportunity to actively discuss wellness and ensure that each resident gets connected with appropriate resources.

## 6 Study strengths and limitations

The study involved participants from both medical schools in the province of Alberta, Canada. The study team collected both quantitative and qualitative data to capture the experiences of the participants through an online survey and semi-structured interviews. Both residents and community preceptors provided rich qualitative insights on the lived realities about the realities of rural residency training. To the authors’ knowledge, this is the first study to examine rural family medicine resident wellbeing through critical realist inquiry with the application of the LSM. One potential limitation is that the study was conducted under extraordinary circumstances during pandemic; however, the lessons learned from this research are transferrable to other situations extenuating from other disruptive events, such as floods and wildfires, which are becoming more common occurrences in Canada and globally. Other limitations of the study may include the small sample size of the study and the contextual specificity during which this study took place, which limits its generalizability. Residents and preceptors who have an interest in wellness may have been more likely to participate in the study which may have introduced bias within the study, although the findings appear to be balanced by both positive and negative factors.

## 7 Conclusion

This study revealed that rural Family Medicine residents’ wellbeing is dynamically constructed across multiple levels of reality and contexts. Using the critical realist lens enabled the authors to map the systemic and relational conditions that shape rural residents’ experiences during both routine training times and disrupted periods. The inherent geographic isolation, resource-strained settings and disruptive events, such as health pandemics and natural disasters, highlight the vulnerability of rural training systems. At the same time, rural identity formation, professional growth and resilience highlight the latent strengths and adaptive capacities of rural residency programs. Residency programs should implement formal wellness and wellbeing initiatives that are responsive, flexible, and grounded in the relational strengths of rural training contexts, including community support and preceptor mentorship.

## Data Availability

The datasets presented in this article are not readily available because of the subject matter (mental health and wellbeing) and nature of clinical placements. Only one or two medical residents are placed in the clinical sites, hence, the interactions and other situational experiences mentioned by the participants in the transcripts could make the individual personalities involved easily identifiable, despite applying great amounts of anonymization. Further inquiries can be directed to the corresponding authors.

## References

[1] Strasser R Neusy A-J. Context counts: training health workers in and for rural and remote areas. Bull World Health Organ. (2010) 88:777–82. doi: 10.2471/BLT.09.072462, 20931063 PMC2947041

[2] Perez AR Boscardin CK Pardo M. Residents’ challenges in transitioning to residency and recommended strategies for improvement. J Educ Perioperative Med: JEPM. (2022) 24:E679. doi: 10.46374/volxxiv_issue1_boscardin, 35707017 PMC9176399

[3] Taher A Hart A Dattani ND Poonja Z Bova C Bandiera G . Emergency medicine resident wellness: lessons learned from a national survey. CJEM. (2018) 20:721–4. doi: 10.1017/cem.2018.416, 30205857

[4] Hirayama Y Khan S Gill C Thoburn M Hancox J Muzaffar J. Enhancing wellbeing in medical practice: exploring interventions and effectiveness for improving the work lives of resident (junior) doctors: a systematic review and narrative synthesis. Future Healthcare J. (2024) 11:100195. doi: 10.1016/j.fhj.2024.100195, 39583992 PMC11584606

[5] Eskander J Rajaguru PP Greenberg PB. Evaluating wellness interventions for resident physicians: a systematic review. J Grad Med Educ. (2021) 13:58–69. doi: 10.4300/JGME-D-20-00359.1, 33680302 PMC7901639

[6] Jennings M Slavin SJ. Resident wellness matters: optimizing resident education and wellness through the learning environment. Acad Med. (2015) 90:1246–50. doi: 10.1097/ACM.0000000000000842, 26177527

[7] Mata DA Ramos MA Bansal N Khan R Guille C Di Angelantonio E . Prevalence of depression and depressive symptoms among resident physicians: a systematic review and meta-analysis. JAMA. (2015) 314:26647259:2373–83. doi: 10.1001/jama.2015.15845 PMC4866499 26647259

[8] Dyrbye L Herrin J West CP Wittlin NM Dovidio JF Hardeman R . Association of racial bias with burnout among resident physicians. JAMA Netw Open. (2019) 2:e197457. doi: 10.1001/jamanetworkopen.2019.7457 31348503 PMC6661712

[9] Slavin SJ Chibnall JT. Finding the why, changing the how: improving the mental health of medical students, residents, and physicians. Acad Med. (2016) 91:1194–6. doi: 10.1097/acm.0000000000001226, 27166866

[10] Dewa CS Loong D Bonato S Thanh NX Jacobs P. How does burnout affect physician productivity? A systematic literature review. BMC Health Serv Res. (2014) 14:325. doi: 10.1186/1472-6963-14-325, 25066375 PMC4119057

[11] Dewa CS Loong D Bonato S Trojanowski L Rea M. The relationship between resident burnout and safety-related and acceptability-related quality of healthcare: a systematic literature review. BMC Med Educ. (2017) 17:195. doi: 10.1186/s12909-017-1040-y, 29121895 PMC5680598

[12] West CP Huschka MM Novotny PJ Sloan JA Kolars JC Habermann TM . Association of perceived medical errors with resident distress and empathy: a prospective longitudinal study. JAMA. (2006) 296:1071–8. doi: 10.1001/jama.296.9.1071, 16954486

[13] West CP Tan AD Habermann TM Sloan JA Shanafelt TD. Association of resident fatigue and distress with perceived medical errors. JAMA. (2009) 302:1294–300. doi: 10.1001/jama.2009.1389, 19773564

[14] Moss SJ Wollny K Amarbayan M Lorenzetti DL Kassam A. Interventions to improve the well-being of medical learners in Canada: a scoping review. CMAJ Open. (2021) 9:E765–76. doi: 10.9778/cmajo.20200236, 34285056 PMC8313096

[15] Edmondson EK Kumar AA Smith SM. Creating a culture of wellness in residency. Acad Med. (2018) 93:966–8. doi: 10.1097/ACM.0000000000002250, 29668521

[16] Sawchuk P. Joys and challenges of rural family medicine. Can Fam Physician. (2019) 65:229. 30867182 PMC6515959

[17] Hodge J. Canadian Healthcare Workers' Experiences during Pandemic H1N1 influenza: Lessons from Canada's Response. Winnipeg, Manitoba, Canada: National Collaborating Centre for Infectious Diseases (NCCID); (2014).

[18] Althwanay A Ahsan F Oliveri F Goud HK Mehkari Z Mohammed L . Medical education, pre-and post-pandemic era: a review article. Cureus. (2020) 12:1–6. doi: 10.7759/cureus.10775, 33154845 PMC7606206

[19] Titus SJ Badar P Coston Z Khan F Ogola GO. COVID-19’s impact on Family medicine Resident Training and Wellness. Baylor University Medical Center Proceedings. San Francisco, California, USA: Taylor & Francis (2023). 10.1080/08998280.2023.2204795 PMC10269402 37334074

[20] Sahu PK Dalçik H. Impact of COVID-19 on Healthcare Professions Education. Dallas, Texas, USA: Frontiers Media SA (2023). p. 1265811. 10.3389/fmed.2023.1265811 PMC10442152 37608825

[21] Boutros P Kassem N Nieder J Jaramillo C von Petersdorff J Walsh FJ . Education and Training Adaptations for Health Workers during the COVID-19 Pandemic: a Scoping Review of Lessons Learned and Innovations. Health. (2023) 11:1–21. doi: 10.3390/healthcare11212902 PMC10649637 37958046

[22] Sherbino J Atzema C. “SARS-Ed”: severe acute respiratory syndrome and the impact on medical education. Ann Emerg Med. (2004) 44:229–31. doi: 10.1016/j.annemergmed.2004.05.021, 15332063 PMC7134708

[23] Patterson D Schmitz D Longenecker R. Family medicine rural training track residencies: risks and resilience. Fam Med. (2019) 51:649–56. doi: 10.22454/FamMed.2019.769343, 31509216

[24] Penwell-Waines L Runyan C Kolobova I Grace A Brennan J Buck K . Making sense of family medicine resident wellness curricula: a Delphi study of content experts. Fam Med. (2019) 51:670–6. doi: 10.22454/fammed.2019.899425, 31269221

[25] Ellaway RH Kehoe A Illing J. Critical realism and realist inquiry in medical education. Acad Med. (2020) 95:984–8. doi: 10.1097/ACM.0000000000003232, 32101916

[26] Pawson R Tilley N. Realistic Evaluation. Philadelphia, Pennsylvania, USA: Lippincott Williams & Wilkins (1997).

[27] Bhaskar R. Forms of realism. Philosophica. (1975) 15:119–112.

[28] Bhaskar R. On the possibility of social scientific knowledge and the limits of naturalism. J Theory Soc Behav. (1978) 8:118–128.

[29] Bhaskar R. A realist Theory of Science. London, England, UK: Routledge (2013).

[30] Ranatunga JB Pagliano PJ. Wellbeing research in education: a critical realist perspective. Int J Innov Creat Chang. (2017) 3:153–72.

[31] Byrne LA How is Wellbeing and the Wellbeing Strategy Defined, Enacted and Experienced in Organisations and Why is this the Case? A Bhaskarian Critical Realist Analysis. Birmingham, England, UK: Ashton University (2023)

[32] Bhaskar R Danermark B Ekstrom M Jakobsen L Explaining Society: An Introduction to Critical Realism in the Social Sciences. London, England, UK: Routledge (2005)

[33] Nunez I. On integral theory: an exercise in dialectical critical realism. J Crit Realism. (2023) 22:431–44. doi: 10.1080/14767430.2023.2217053

[34] Jackson AY Mazzei LA. Plugging one text into another: thinking with theory in qualitative research. Qual Inq. (2013) 19:261–71. doi: 10.1177/1077800412471510

[35] Braun V Clarke V. Conceptual and design thinking for thematic analysis. Qual Psychol. (2022) 9:3. doi: 10.1037/qup0000196

[36] Collins CS Stockton CM. The central role of theory in qualitative research. Int J Qual Methods. (2018) 17:1609406918797475. doi: 10.1177/1609406918797475

[37] Checkland K. The myth of the emerging themes: why qualitative research needs theory. Br J Gen Pract. (2024) 75:246. doi: 10.3399/bjgp25X742461 PMC12117625 40441906

[38] Crabtree BF Miller WL, editors. “Doing qualitative research.” *Annual North American Primary Care Research Group Meeting, 19th, May, 1989, Quebec, PQ, Canada*; (1992): Sage Publications, Inc.

[39] Braun V Clarke V. One size fits all? What counts as quality practice in (reflexive) thematic analysis? Qual Res Psychol. (2020) 18:328–52. doi: 10.1080/14780887.2020.1769238

[40] Kassam A Ellaway R. Acknowledging a holistic framework for learner wellness: the human capabilities approach. Acad Med. (2020) 95:9–10. doi: 10.1097/ACM.0000000000003026, 31860619

[41] Government of Alberta - Canada. Rural health professions action plan. (2024). Available online at: https://rhpap.ca/ (Accessed February 19, 2026).

[42] Nohales L Fort E Pelloux S Coste C Leblanc P De Ternay J . Occupational, academic, and personal determinants of wellbeing and psychological distress in residents: results of a survey in Lyon, France. Front Psychol. (2024) 15:1347513. doi: 10.3389/fpsyg.2024.1347513, 38770261 PMC11103015

[43] McKenna KM Hashimoto DA Maguire MS Bynum WE IV. The missing link: connection is the key to resilience in medical education. Acad Med. (2016) 91:1197–9. doi: 10.1097/ACM.0000000000001311, 27438155

[44] Kelly M Perez G Ram R Begert N Keshvara A Johnston A. Becoming more integrated into the community: a qualitative study of learners' experiences of the learning environment in a longitudinal integrated clerkship. Front Med. (2025) 12:1609051. doi: 10.3389/fmed.2025.1609051, 40718427 PMC12290413

[45] Toubassi D Schenker C Roberts M Forte M. Professional identity formation: linking meaning to well-being. Adv Health Sci Educ. (2023) 28:305–18. doi: 10.1007/s10459-022-10146-2, 35913664 PMC9341156

[46] Wald HS. Professional identity (trans) formation in medical education: reflection, relationship, resilience. Acad Med. (2015) 90:701–6. doi: 10.1097/ACM.0000000000000731, 25881651

[47] Kassam A Martimianakis MA. When I say… wellness. Med Educ. (2024) 58:380–1. doi: 10.1111/medu.15297 38093703

