# Supplementary material for: Navigating rural medical training: mapping the landscape of resident physician wellbeing using critical realist inquiry
Source: Front Med (Lausanne). 2026 Mar 30;13:1792445. doi: 10.3389/fmed.2026.1792445 (PMC13072648; doi:10.3389/fmed.2026.1792445)
Supplement: Supplementary file 1 [file Data_Sheet_1.pdf]

## Resident Interview Guide

**Interview Questions**

1. Why did you decide to take part in a rural placement as part of your residency?
    a. Did you have any reservations or concerns about the placement?
    b. Did you have any rural experience before starting the placement? If so, describe.

2. What was the process of moving to [rural placement location] like for you?
    a. Did you receive financial support from the university or program?
    b. Did you encounter any issues looking for housing?

3. What has your experience of living in [rural placement location] been like?
    a. Do you feel as if you have integrated into the community? Why not or in what way?
    b. Did you experience any feelings of isolation (social or professional)?
        - If so, how did you manage those feelings? Were there any supports or resources available for you to access?
    c. Do you take part in any community activities or use any community resources? (ie. recreational centers, sports, religious or spiritual centers etc).
        - Do the community amenities meet your needs?
    d. How has COVID-19 impacted your experience of living in [rural placement location]?

4. Do you have experience practicing in an urban environment? If so, have you observed any differences in your urban placement versus your rural placement?
    a. Have you observed any differences in the professional or academic support you have received? If so, how have you managed that?
    b. Have you observed any differences in the workload?
        - Has that affected your stress levels or overall mental health? How have you managed that? What resources have you accessed?
        - Did you complete your rural placement during the COVID-19 pandemic? If so, how did the pandemic impact your stress levels and mental health? How did the pandemic change how you managed your stress and mental health? How did the pandemic impact the resources you accessed?

5. [For participants who have only had rural experiences]
    a. How would you describe the professional or academic support you have received?
    b. What is the workload of your rural residency like?
        - Have you observed any changes in your stress level or overall mental health since beginning your residency?
            - How have you managed that? What resources have you accessed, or are you aware of any resources for residents in this regard?
            - Are there any informal networks that you are able to access or that you are aware of?
        - How has COVID-19 impacted your stress level or mental health? How has the pandemic changed how you manage your stress and mental health? How has the pandemic impacted the resources you access?

6. Based on your experiences in your rural residency placement, would you choose to establish a practice in a rural area? Why or why not?
    a. [If yes] have you taken any steps towards establishing a practice in a rural area?

7. Would you recommend rural residency for a student considering it? Why or why not?
    a. What advice would you give to a student preparing for rural residency?

8. Do you have anything else to add or elaborate on about your rural placement or residency, or your experience in the rural community?

*Conclusion and Wrap Up*

Thank you, that's all the questions we have for today. Thank you for taking the time to speak with me [turn off recorder].
