# Supplementary material for: Navigating rural medical training: mapping the landscape of resident physician wellbeing using critical realist inquiry
Source: Front Med (Lausanne). 2026 Mar 30;13:1792445. doi: 10.3389/fmed.2026.1792445 (PMC13072648; doi:10.3389/fmed.2026.1792445)
Supplement: Supplementary file 2 [file Data_Sheet_2.pdf]

Preceptor Interview Guide

Interview Questions

1. Can you describe what role or interaction you have with residents at your rural site?
    a. Does your role include a mentorship aspect? In what way?
    b. What sort of academic or professional support do you provide for residents at your site?
    c. What sort of personal support do you provide for residents at your site?

2. Can you describe the wellness concerns you have observed in residents training at your rural site?
    a. What type of mental health/housing/community integration/academic and/or professional support concerns have you noticed in the residents at your site?
    b. Have you observed any differences in the wellness issues students face depending on the type or length of placement?
    c. [If practiced in an urban setting] Have you observed any differences in the wellness issues that rural vs urban students face?

3. Has a resident ever approached you with a wellness concern?
    a. What types of wellness concerns have you been approached about?
    b. Is there a formal procedure for residents to seek help with wellness concerns?
    c. What, if any, support or resources do you refer students to in these situations?
        i. Are there any informal support networks you are aware of?
        ii. What supports are available in the community that you might refer a resident to?

4. What resources are available at your clinical site to address resident wellness concerns?
    a. Do you feel as if these resources adequately address the resident wellness concerns you have observed?
    b. Are there any gaps in the resources?
    c. In what ways could support for rural residents be improved at your clinical site?

5. How has COVID-19 impacted the wellness concerns you have observed in residents? How has it impacted the resources available to residents at your clinical site?

6. Do you have anything else to add or elaborate on about rural resident wellness at your clinical site?

Conclusion and Wrap Up

Thank you, that’s all the questions we have for today. Thank you for taking the time to speak with me [turn off recorder].
